# Polycyclic Aromatic Hydrocarbon (PAH) Exposure and DNA Adduct *Semi*-Quantitation in Archived Human Tissues

**DOI:** 10.3390/ijerph8072675

**Published:** 2011-06-29

**Authors:** M. Margaret Pratt, Kaarthik John, Allan B. MacLean, Senait Afework, David H. Phillips, Miriam C. Poirier

**Affiliations:** 1 Carcinogen-DNA Interactions Section, National Cancer Institute, Bldg 37, Rm 4032 NIH, 37 Convent Drive, Bethesda, MD 20892, USA; E-Mails: kaarthikj@gmail.com (K.J.); poirierm@exchange.nih.gov (M.C.P.); 2 Department of Obstetrics and Gynaecology, Royal Free Hospital Hampstead, Pond Street, London, NW3 2QG, UK; E-Mails: a.maclean@ucl.ac.uk (A.B.M.); senuch@yahoo.com (S.A.); 3 Section of Molecular Carcinogenesis, Institute of Cancer Research, Brookes Lawley Building, Cotswold Road, Sutton, SM2 5NG, UK; E-Mail: d.phillips@qub.ac.uk

**Keywords:** DNA damage, human tissues, immunohistochemistry, immunoassay, PAH-DNA adducts, *semi*-quantitation, molecular epidemiology

## Abstract

Polycyclic aromatic hydrocarbons (PAHs) are combustion products of organic materials, mixtures of which contain multiple known and probable human carcinogens. PAHs occur in indoor and outdoor air, as well as in char-broiled meats and fish. Human exposure to PAHs occurs by inhalation, ingestion and topical absorption, and subsequently formed metabolites are either rendered hydrophilic and excreted, or bioactivated and bound to cellular macromolecules. The formation of PAH-DNA adducts (DNA binding products), considered a necessary step in PAH-initiated carcinogenesis, has been widely studied in experimental models and has been documented in human tissues. This review describes immunohistochemistry (IHC) studies, which reveal localization of PAH-DNA adducts in human tissues, and *semi*-quantify PAH-DNA adduct levels using the Automated Cellular Imaging System (ACIS). These studies have shown that PAH-DNA adducts concentrate in: basal and supra-basal epithelium of the esophagus, cervix and vulva; glandular epithelium of the prostate; and cytotrophoblast cells and syncitiotrophoblast knots of the placenta. The IHC photomicrographs reveal the ubiquitous nature of PAH-DNA adduct formation in human tissues as well as PAH-DNA adduct accumulation in specific, vulnerable, cell types. This *semi*-quantative method for PAH-DNA adduct measurement could potentially see widespread use in molecular epidemiology studies.

## 1. Chemical Carcinogenesis and DNA Adduct Formation

Human DNA adducts, as biomarkers of exposure, internal dose and biologically effective dose, are capable of initiating mutagenesis in critical genes, ultimately leading to a loss of growth control followed by tumor development [1–3]. Environmental monitoring documents the presence of a xenobiotic agent, but the biological consequences of exposure to the organism are found only intracellularly [4,5]. Protein-adduct formation is considered a surrogate of DNA adduct formation, but only the latter results in critical mutagenic changes [6]. Multiple studies in experimental models [7,8] have shown that DNA adduct formation is associated in a dose-related fashion with tumor induction. In addition, a number of epidemiological investigations have demonstrated an association between DNA adduct formation and increased human cancer risk [9,10]. Therefore, DNA adducts are considered to be necessary but not sufficient for tumor induction. Furthermore, reduced DNA adduct formation in experimental models has been associated with reduced tumor incidence [11–13], suggesting that, if DNA damage in humans could be reduced, cancer risk might also be attenuated.

## 2. Human Exposure to Polycyclic Aromatic Hydrocarbons (PAHs)

Human exposure to combustion products and mixtures containing carcinogenic PAHs has long been associated with cancer induction [14]. Over 200 years ago it was reported that chimney sweeps, who often worked unclothed and bathed rarely, were highly susceptible to cancer of the scrotum. Subsequently, bathing as a deterrent to cancer induction became an early successful intervention, confirming the association between soot exposure and scrotal cancer risk [3]. By the late 1970s PAH-DNA adduct formation was well documented in experimental models, primarily through the use of radiolabeled compounds. Non-invasive methods to measure DNA damage in human tissues had yet to be developed [15]. Investigators recognized that in order to extend knowledge of mechanistic cancer etiology in humans it would be necessary to develop highly-sensitive, non-invasive methods of DNA adduct measurement. The development of antisera specific for carcinogen-DNA adducts or carcinogen-modified DNA was one such approach that allowed for DNA adduct determination using immunohistochemistry of archived tissue as well as immunoassay of extracted DNA [16].

## 3. Use of Immunoassay and Immunohistochemistry for Human PAH-DNA Adduct Detection

To examine PAH-DNA adducts in human tissues, investigators elicited a rabbit antiserum against DNA modified with an activated form of benzo[*a*]pyrene (BP), the r7,t8-dihydroxy-t-9, 10-oxy-7,8,9,10-tetrahydrobenzo[*a*]pyrene (BPDE) [17]. The immunogen DNA contained a single adduct, the r7,t8,t9-trihydroxy-c-10-(N^2^deoxyguanosyl)-7,8,9,10-tetrahydrobenzo[*a*]pyrene (BPdG) adduct. However, the resulting antiserum cross-reacted with DNA samples modified with diol-epoxides of additional PAHs including: chrysene, benzo[*k*]fluoranthene, dibenz[*a,c*]anthracene, and the bay-region and non-bay region benz[*a*]anthracenes [18]. Individually, these compounds are carcinogenic in animal models and have been classified by the US Environmental Protection Agency (US EPA) and the International Agency for Research on Cancer (IARC) as either known or probable human carcinogens [19]. Because most human PAH exposures are to complex mixtures, human DNA samples are presumed to contain multiple PAH-DNA adducts, many of which will be detected by the BPDE-DNA antiserum. The resulting increased sensitivity may be advantageous for biomonitoring and measuring DNA damage induced by a family of carcinogenic PAHs. Where nomenclature is concerned, the BPDE-DNA immunoassays measure only BPdG in samples from animals exposed experimentally to BP, but when used with human samples the results are termed “PAH-DNA adducts” because human DNA likely contains adducts of multiple carcinogenic PAHs.

### 3.1. Validation of PAH-DNA Measurements by Immunoassay

Over the years many studies have contributed to validation of PAH-DNA adduct measurements in human DNA using immunoassays, the most-sensitive of which is the chemiluminescence immunoassay (CIA) [20]. These studies have shown that human blood cell PAH-DNA adducts result from dietary ingestion of PAHs [21], increase or decrease as a function of the quantity of PAHs ingested [22], and have a half-life of about a week in human blood [23–25]. In wildland firefighters, variations in blood cell PAH-DNA adducts correlated with recent charbroiled meat consumption and not recent firefighting activity [24,25]. When PAH levels in the diet are low, inhalation can be a significant source of PAH exposure. A cohort study of US Army soldiers who were moved from an area with a clean ambient environment to one that was much more polluted revealed significant increases in blood cell PAH-DNA adduct levels for each individual [26,27]. Similarly, a significantly higher level of blood cell PAH-DNA adducts was found in young adults sampled in Mexico City in the dry season, when airborne PAH levels were higher, compared to the rainy season, when PAH levels were lower [28]. Using samples from the Navy Colon Adenoma study we found a 3-fold increased risk of colorectal adenoma in the quartile of individuals with the highest leukocyte PAH-DNA adducts, compared to the quartile with the lowest PAH-DNA adducts [29]. These were, coincidentally, the same individuals who consistently ingested the largest quantities of heavily-cooked beef [30], suggesting a causal association between ingestion of well-cooked beef, PAH-DNA adduct formation and adenoma risk. Taken together, all of the above studies suggest that PAH-DNA adduct levels vary with PAH exposures, and that reducing PAH-DNA levels might also decrease human cancer risk.

### 3.2. Localization and Semi-Quantitation of PAH-DNA Adducts by Immunohistochemistry (IHC)

We have had a long-term interest in the use of IHC for localization of PAH-DNA adducts using the same antiserum employed for immunoassays (above). Using IHC, tissue morphology is retained and there is the potential to reveal “target” cells, those having the highest concentration of DNA adducts. In addition, molecular epidemiology studies require large numbers of samples, and archived paraffin blocks are a ready source of such materials. In this review we will describe studies using the BPDE-DNA antiserum to localize and *semi*-quantify PAH-DNA adducts in multiple human tissues, including esophagus, prostate, cervix, vulva, and placenta.

All of the IHC studies to be described here involved automated staining (using Ventana NexES) of paraffin-embedded tissues with the BPDE-DNA antiserum, and the use of a Fast Red—conjugated secondary antiserum to reveal adducted (pink) nuclei [31]. Hematoxylin staining of an adjacent tissue section revealed the total number of nuclei in the area chosen for evaluation, and the staining of an additional adjacent tissue section with BPDE-DNA antiserum absorbed with the immunogen, BPDE-DNA, provided a negative control, which demonstrated the specificity of the PAH-DNA signal. The Automated Cellular Imaging System (ACIS; Chromavision, Inc.), which has the capacity to automate, digitize and expedite pathology evaluations [32,33], was used to score DNA adducts in tissue nuclei. Following capture of a high-resolution digital image, operator-defined regions were evaluated according to study-specific parameters of color/hue (red-pink) and object/nucleus size and shape. The resulting ACIS-discerned nuclear color intensity was reported as Optical Density (OD). Identical regions in adjacent hemotoxylin-stained sections were evaluated to provide a count of nuclei for each respective region. The resulting OD/nucleus values were then converted to units of adducts/10^8^ nucleotides by comparing the unknown samples to a standard curve.

**Figure 1:**
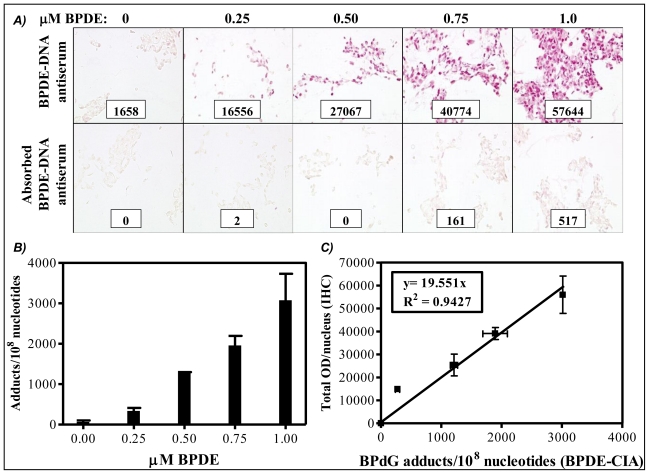
Standard curve consisting of cultured human keratinocytes exposed to 0, 0.25, 0.50, 0.75, or 1.00 μM BPDE for 1 h.

Cultured human keratinocytes exposed to incremental concentrations of BPDE were separated into two portions. Half of the BPDE-exposed human cells were fixed in formalin, embedded in paraffin then stained for PAH-DNA adducts by IHC (Figure 1A). Cells stained with specific and immunogen-absorbed antiserum are shown, respectively, in the top and bottom rows of Figure 1A. Inserts contain staining intensity values (OD/nucleus) for the pink color determined by ACIS. DNA was extracted from the remaining BPDE-exposed human cells and quantitatively analyzed for BP-DNA adducts by BPDE-DNA CIA (Figure 1B). The standard curve (Figure 1C) comprises a graph in which OD/nucleus values (Figure 1A) are plotted as a function of the BPdG adducts/10^8^ nucleotides values determined by CIA (Figure 1B). Values represent mean ± range for two CIAs (each with triplicate wells), and mean ± range for two IHC arrays, with staining of three cores and three regions/core for each experimental group (Adapted from John, *et al*., 2009 [34]). The resulting values for PAH-DNA adducts/10^8^ nucleotides in unknown samples are *semi*-quantitative because of the uncertain composition of PAH adducts in human DNA. In addition, the numbers generated for PAH-DNA adducts/10^8^ nucleotides by IHC/ACIS are substantially higher than those measured when DNA is extracted from whole tissue and adducts are measured by BPDE-DNA CIA. This occurs largely because the IHC methodology allows us to choose a specific, highly-modified target area for evaluation, whereas, for CIA measurements, the extracted DNA is from all cells in the sample tissue.

## 4. Human Esophagus

**Figure 2:**
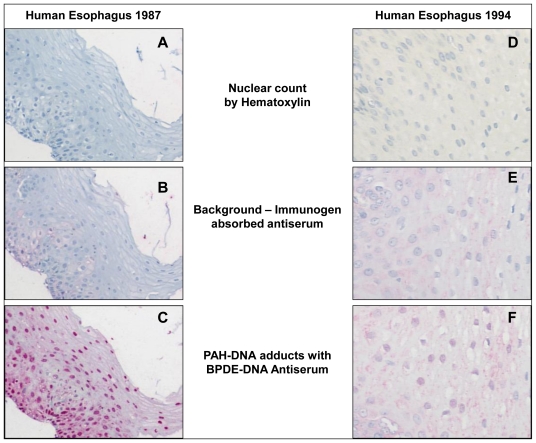
Human esophageal biopsy samples collected in 1987 and 1994 then stained and analyzed up to 17 years later by BPDE-DNA IHC/ACIS.

Linxian (Linxou), China, is located in a mountainous region where indoor coal stoves lacking external venting are used for cooking and heating and the cumulative mortality rate from esophageal and proximal stomach cancer is among the highest in the world. In this area, high levels of carcinogenic PAHs, including BP, have been detected in raw and cooked food [35]. The tissues examined in our study were biopsies collected from patients for the purpose of diagnosing squamous carcinoma-*in situ* or early invasive squamous cell carcinoma. The samples were obtained and paraffin-embedded in 1985 [31] and 1994 [36]. Because of the age of the samples, we discarded the first several cuts off each paraffin block in order to perform the staining on non-oxidized tissue. Figure 2 shows samples of human esophagus taken from Linxian in 1985 and 1994 and stained up to 17 years later with: hematoxylin (Figure 2A and D); immunogen-absorbed BPDE-DNA antiserum (Figure 2B and E, shows specificity of antiserum); and specific BPDE-DNA antiserum (Figure 2C and F). In Figure 2C it is evident that PAH-DNA adducts concentrate in the nuclei of the esophageal epithelium, especially in the vicinity of the basal cell layer. For comparison, and to validate the methodology, esophageal tissue was collected at autopsy from 6 individuals in the US who died from conditions unrelated to esophageal cancer. Staining of these tissues (not shown) revealed no evidence of PAH-DNA adduct formation [31]. Taken together, these studies indicate that tissues archived for many years can successfully be queried for the presence of PAH-DNA adducts as exposure biomarkers. In addition, it appears that the population of Linxian is exposed to PAHs through the ambient air and the food supply, and both sources of exposure likely contribute to the observed PAH-DNA adducts. The presence of PAH-DNA adducts in esophagus is consistent with the epidemiological finding that esophageal tumor incidence is high, however, a causal association between PAH exposure, PAH-DNA adduct formation and esophageal cancer risk remains to be established.

## 5. Human Prostate

**Figure 3:**
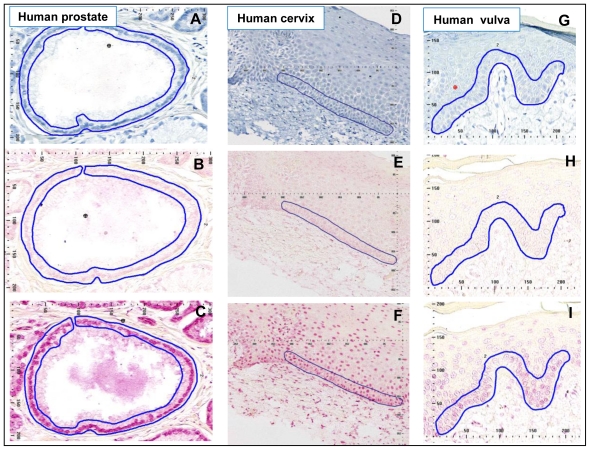
Representative human prostate, cervix and vulvar tissue biopsies evaluated for PAH-DNA adducts*. * Tissues stained with hematoxylin (A, D and G, respectively) to obtain a count of total nuclei; immunogen (BPDE-DNA)-absorbed serum (B, E and H, respectively) to demonstrate specificity of the serum; and specific BPdG-DNA serum (C, F and I, respectively) to show the presence of PAH-DNA adducts. The tic marks indicate 10 μm intervals and are for the purpose of image localization.

The etiology of prostatic adenocarcinoma is interesting in that the tumors arise almost exclusively in the peripheral zone (PZ) of the prostate gland, whereas the transition zone (TZ) tissue develops occasional benign hypertrophy, but no tumors. In Western countries an increased risk of adenocarcinoma of the prostate is associated with diet and lifestyle factors, including smoking. Therefore, we hypothesized that if PAH-DNA adduct formation played a role in prostate cancer etiology, the PZ might have higher levels of adducts than the TZ. To investigate this we examined PAH-DNA adduct formation in non-tumorous portions of prostate tissue collected from 23 men undergoing either radical retropubic prostatectomy for low-volume prostatic adenocarcinoma (n = 22) or cystoprostectomy for bladder cancer (n = 1) [34]. The patients were largely working-class, middle-aged men living in an industrialized area of the UK, with the majority reported to be smokers. Metabolic capacity, determined by expression studies of xenobiotic metabolizing enzymes, showed essentially similar metabolic capacity in PZ compared to TZ [34], and normal appearing PZ and TZ areas were sectioned and examined by IHC/ACIS for PAH-DNA adducts (Figure 3A–C).

Regardless of which zone was evaluated, the PAH-DNA adducts were localized primarily in the glandular epithelium (Figure 3C). Overall, there was no significant difference in levels of PAH-DNA adducts localized in the PZ and the TZ, despite stratification by smoking status and diet. Using the standard curve shown in Figure 1, PAH-DNA adduct values ranged from 8 adducts/10^8^ nucleotides (the limit of detection, LOD), up to 1,812 and 2,214 adducts/10^8^ nucleotides in the PZ and TZ, respectively [34]. These were the highest values, and the most intensely-stained nuclei observed in any human tissue using the IHC/ACIS methodology (Table 1). In fact, these values were approximately 10-fold higher than the PAH-DNA adducts observed using the same staining method for human cervix, vulva and placenta (Table 1). We conclude that all zones of the human prostate sustain essentially similar PAH-DNA damage, which is primarily localized in the ductal epithelium. The particular susceptibility of the PZ to form tumors may result from the influence of PAH-DNA damage as well as other types of DNA damage and additional factors such as increased cell proliferation, inflammatory stimulation and epigenetic alterations. This study shows that prostate tissue is prone to high levels of PAH-DNA damage, but suggests that additional factors contribute to prostate cancer risk.

## 6. Human Cervix

In women infected with carcinogenic strains of the human papilloma virus (HPV), current and past smoking has been associated with a significantly increased risk of developing cervical cancer. We hypothesized that individuals with the higher concentrations of PAH-DNA adducts localized in the cervix may have the highest cancer risk, and that adduct formation would be associated with the womens’ smoking status. PAH-DNA adducts were evaluated in cervix tissue collected from HPV-infected women as part of a 10-year prospective study of factors that may increase the risk of developing cervical cancer [37,38]. We stained 78 coded samples, later revealed to consist of 29 cancer cases and 49 controls, and of these, 75 samples had sufficient normal-appearing epithelial tissue for IHC/ACIS analysis (Figure 3D–F) [38]. Localization of PAH-DNA adducts occurred throughout the cervix epithelium (Figure 3F), though intensity of staining appeared to fade in the uppermost regions of the mature squamous epithelium. The largest nuclei and the highest PAH-DNA adduct concentration appeared in the basal layer, the region which was chosen for analysis (Figure 3F). An average of 415 cells was counted for each sample and, using the standard curve (described above), the calculated PAH-DNA adduct values ranged from 20 (the LOD) to 191 adducts/10^8^ nucleotides (Table 1).

The data revealed a lack of correlation between DNA adduct formation and risk of cancer of the cervix. In addition, no correlation was found between PAH-DNA adduct formation and smoking status, stratified as never, former, low current (<10 cigarettes/day) and high current (>10 cigarettes/day) smoking. Unfortunately, the stratified sample groupings were small, and the demographic data did not reveal details of diet, second-hand smoke, or occupational PAH exposure. This study showed that PAH-DNA adducts are formed in human cervix with substantial (10-fold) interindividual variability, and are primarily localized in the basal cell layer of the cervix epithelium. The lack of a smoking correlation supports the notion that PAH-DNA damage formation, as determined by this staining approach, is not primarily a result of smoking. Literature data indicate that the alternate sources of PAH-exposure likely include ambient pollution, other inhaled products of organic combustion, and diet. The data presented here also suggest that carcinogenic components of cigarette smoke, other than the PAHs, may be responsible for the smoking-associated cancer risk in women infected with carcinogenic strains of HPV. Additional studies will be required to elucidate the relationship between smoking and cancer of the cervix.

## 7. Human Vulva

**Figure 4:**
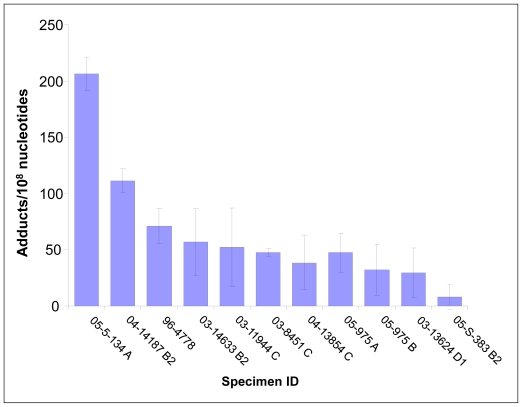
PAH-DNA adducts/10^8^ nucleotides (mean ± SD) as determined by IHC/ACIS in 3–4 different areas of vulvar basal epithelium in each of 10 individuals.

In a small pilot study, a series of human vulva biopsy samples, taken from non-involved areas, was obtained from 10 women being treated for vulvar intraepithelial neoplasia (VIN). We expected to find levels of PAH-DNA adducts in vulva similar to those observed in the cervix. Samples were evaluated by BPDE-DNA IHC/ACIS and comparison was made with the standard curve. The highest PAH-DNA adduct concentrations were found in the basal cells of the vulvar epithelium (Figure 3G–I), similar to the localization observed in human cervix. All of the women examined had measurable PAH-DNA adducts, with values ranging from 8 (the LOD) to 206 adducts/10^8^ nucleotides (Figure 4). The vulva is susceptible to cancerous changes, which are less prevalent but otherwise not unlike those found in cervix. This pilot study demonstrated that PAH-DNA adducts are formed in vulvar epithelium (Table 1), but a much larger study will be required to examine the contribution of PAH-DNA damage to risk of VIN.

## 8. Human Placenta

**Figure 5:**
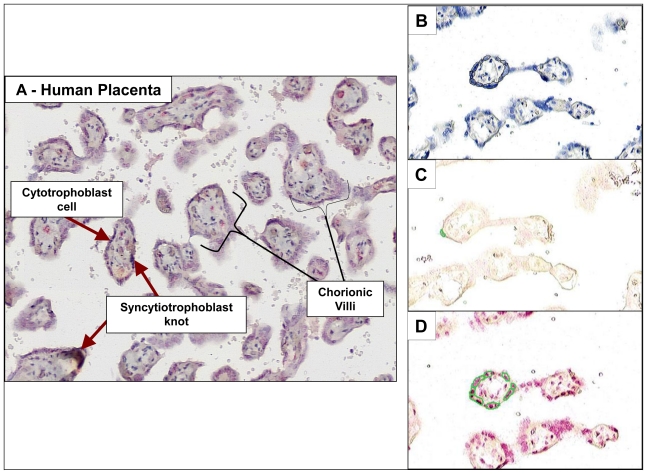
Overview of human placenta tissue architecture and example of scored tissue region.

The placenta is not ordinarily considered a tissue subject to cancer risk, but it is a tissue of interest as an indicator of maternal and fetal exposure to carcinogens during pregnancy. We examined placenta samples obtained at birth following normal, full-term pregnancies in Teplice, an area of the Czech Republic historically known to have high levels of airborne pollution. In a long-term series of studies [39,40], children born in Teplice were found to have multiple health problems including intrauterine growth retardation, low birth weight and respiratory ailments. The object of our study was to determine PAH-DNA distribution in different cell types of the placenta and, if successful, to attempt quantification of the damage. We hypothesized that the metabolically-active cells would have the highest concentrations of PAH-DNA adducts. Placenta samples from seven smokers and seven non-smokers were stained and evaluated by IHC/ACIS [41]. PAH-DNA adducts were concentrated in the cytotrophoblast cells and synctiotrophoblast knots lining the chorionic villi, finger-like projections which reach into the maternal blood (Figure 5A). These cells, which are of fetal origin, are capable of metabolizing xenobiotics and it was, therefore, not a surprise that they contained highest concentrations of PAH-DNA adducts. Figures 5B–D shows formalin-fixed paraffin-embedded human placenta samples collected and preserved immediately following normal, full-term pregnancies of women living in the Teplice region of the Czech Republic. Adjacent, parallel sections from a single placenta were stained with (B) hematoxylin, to obtain a count of total nuclei, (C) immunogen (BPDE-DNA)-absorbed serum, to demonstrate specificity of the serum, and (C) BPDE-DNA specific serum showing the presence of PAH-DNA adducts. The small circled areas indicate the ACIS-identified objects/nuclei within user-specified regions of interest.

By comparison with the BPDE-exposed human cervical keratinocyte standard curve, the level of PAH-DNA adducts in the placenta samples ranged from 49 to 312 PAH-DNA adducts/10^8^ nucleotides, well above the LOD of 20 adducts/10^8^ nucleotides [41]. There was no significant difference in PAH-DNA adduct levels between smokers and non-smokers. However, the highest PAH-DNA adduct concentration found in placentas from Teplice, 312 adducts/10^8^ nucleotides, was 1.5-fold higher than the highest levels found in human cervix and vulva (Table 1) and consistent with the reports of high levels of ambient pollution in Teplice. We conclude that the metabolically competent cells of the Teplice placentas sustained high levels of PAH-DNA damage, reflective of significant PAH-exposures. The lack of char-broiled meat or fish in the local diet suggests that the primary source of this exposure may be from ambient air.

## 9. Methodological Considerations

The validity of this method as an indicator for cancer risk deserves mention. The antiserum is not specific for DNA damage induced by an individual PAH, but recognizes DNA adducts induced by a family of PAHs, many of which are either animal or human carcinogens, or both. Because most hydrocarbon exposures occur as complex mixtures, humans are exposed to everything contained in those mixtures. The BPDE-DNA antiserum detects DNA damage induced by several PAH carcinogens. Though less likely to form DNA adducts, this assay likely recognizes DNA damage induced by some non-carcinogens as well. Given that complex PAH mixtures typically contain PAH carcinogens, our assumption is that a positive PAH-DNA signal measured by the BPDE-DNA antiserum indicates a potential cancer-associated exposure. The PAH-DNA values generated by IHC/ACIS are *semi*-quantitative, and the numbers generated are 10–100 fold higher than those observed with BPDE-DNA CIA using extracted DNA. It is important to note, however, that IHC/ACIS values reflect investigator-selected areas, which are typically regions having the highest PAH-DNA adduct color intensity, whereas extraction of DNA from all the cells in a tissue results in effective dilution, as PAH-DNA adducts are not uniformly distributed to all tissues.

Several important technical challenges arose as these studies progressed. For example, in a complex tissue where adduct intensity may vary between cell types, it is important to decide which features of tissue architecture (*i.e*., basal epithelium) will be examined so that consistency can be maintained throughout the analysis of a single group of samples. Also, it is important to evaluate cells in multiple (at least three or four) areas of a tissue section to incorporate as much of the intra-tissue variability as possible. In performing the placenta study [41] we learned by experience that fresh tissue should be fixed and embedded immediately, and never frozen before paraffin blocks are made. The Linxian study [31,36] taught us that sectioned tissue on slides will oxidize over time, and it is therefore important to use fresh-cut tissue from an interior area of the paraffin block, not the first sections cut after storage of the block. Also, it is best to section the tissues no more than a couple of months before staining. Embedding the whole standard curve into a single block as a tissue array, so it can be placed on a single slide and stained simultaneously, has been a highly successful strategy that we owe to our collaborator, Stephen Hewitt [42]. In addition, for the most accurate comparison, the standard curve array should be sectioned and stained at the same time as the samples to be evaluated.

## 10. Additional IHC Studies with Human Tissues

The studies presented here have been described in detail because they were all performed in the same lab using the same antiserum, staining process and image analysis. In addition, these studies employed the same type of Standard Curve that allowed *semi*-quantitation of PAH-DNA adduct values, and therefore the data from different studies could be compared (Table 1). However, similar types of DNA adduct *semi*-quantitation have been reported for other human tissues during investigations of a variety of carcinogens. Additional types of DNA damage evaluated in human tissues by IHC with image analysis include DNA adducts induced by: 4-aminobiphenyl [43,44]; 2-amino-1-methyl-6-phenylimidazole[4,5-b]-pyridine (PhIP) [45]; malondialdehyde [46]; and endogenous oxidative stress [47]. PAH-DNA adducts have been detected by IHC in human exfoliated oral cells and urothelial cells [48,49] in studies comparing PAH-DNA adducts in smokers and non-smokers. The smokers were recruited from the New York City area [49] and from Rome, Italy [48], and both studies showed that smokers had higher levels of PAH-DNA adducts in exfoliated cells than non-smokers. Taken together, all the IHC studies support the ubiquitous nature of DNA adduct formation in human tissues, as PhIP-DNA adducts and 2-aminobiphenyl-DNA adducts were found in normal human breast [43,45], 1,*N^6^*-ethenodeoxyadenosine was found in diseased human liver [47], and several types of adducts were found in exfoliated oral and urothelial cells [44,46,48]. Information regarding DNA adduct distribution in human tissues and cells elucidates human exposure and may inform decisions related to prevention and management of cancer risk.

## 11. Conclusions and Future Perspectives

Several important conclusions can be drawn from these studies, the data for which are summarized in Table 1. *First*: Environmental and dietary PAH exposures result in PAH-DNA adduct formation simultaneously in many tissues of the body. *Second*: PAH-DNA adducts in human tissues are concentrated in organ-specific cell types, a phenomenon which results in higher DNA adduct values determined by IHC/ACIS compared to conventional immunoassay analysis where DNA is extracted from all the cells of a tissue. *Third*: These studies confirm a broad inter-individual range (10- to 20-fold) for levels of PAH-DNA adducts in a single tissue type from a single cohort. *Fourth*: IHC/ACIS of archived human tissues may be an attractive approach for PAH-DNA adduct evaluation as paraffin blocks are readily available and the current studies show these adducts to be stable in paraffin for at least 20 years. *Fifth*: Whereas the assay is only *semi*-quantitative, the standard curve stained concurrently with the tissue sections allows comparison of tissues stained at different times, suggesting a consistency in the method that reveals real differences between tissues (Table 1). For example, it is possible that the high PAH-DNA levels in prostate indicate high levels of exposure in the population studied, but it is also possible that the prostate is particularly susceptible to this type of DNA damage.

Evaluation of the biologically effective dose for PAH exposure, through detection of PAH-DNA adducts, is an important step forward in our understanding of the connection between chemical exposure and cancer development, since cellular events are necessarily more relevant to disease outcome than measurement of PAH levels in the ambient environment or diet. Quantifying PAH exposures by monitoring intake is informative, but does not account for interindividual metabolic variations, which we and others have shown to be substantial. Nor does it reveal tissue-specific differences, which are important since it is evident that not all tissues are targets for tumor formation. In fact, in esophagus, cervix and vulva, observations that PAH-DNA adducts concentrate in the epithelial basal cell layer are consistent with the susceptibility of these cells to tumor induction.

The ability to assess biomarkers of exposure in archived whole tissue will allow for: generation of large quantities of data; new information on cellular processing of DNA damage; and comparison of PAH-DNA information with additional methodologies to elucidate mechanism. Availability of new specialized techniques, such as laser capture microdissection, may provide additional opportunities to collect and evaluate very specific portions of whole tissue, or individual cells, in which PAH-DNA damage can potentially be related to other molecular markers such as gene expression, micro-RNAs or epigenetics.

## Figures and Tables

**Table 1:** Comparison of PAH-DNA adduct values in human tissues by IHC/ACIS.

Tissue	Number of Subjects	PAH-DNA adducts/10^8^ nucleotides				Cell type with highest PAH-DNA Adducts
		Non-detect subjects	Low	High	Cells/subject	
Esophagus	10	1	NA^a^	NA	1,000–8,000	Basal epithelial
Prostate	23	2	8^b^	2,214	200–300	Ductal epithelium
Cervix	75	3	20^b^	191	72–2,200	Basal epithelial
Vulva	10	1	20^b^	206	120–747	Basal epithelial
Placenta	14	0	49	312	200–400	Cytotrophoblast and Syncytiotrophoblast

a NA = not assayed; the standard curve quantitation was not performed with the esophagus samples, but was performed with the prostate, cervix, vulva and placenta samples.

b Shows the LOD for each assay. The LOD for the placenta study was 20 adducts/10^8^ nucleotides but the lowest human value was 49.

## Abbreviations

ACIS: Automated Cellular Imaging System
BP: benzo[*a*]pyrene
BPDE: r7, t8-dihydroxy-t-9, 10-epoxy-7, 8, 9, 10-tetrahydrobenzo[*a*]pyrene
BPdG: r7, t8, t9-trihydroxy-c-10-(N^2^deoxyguanosyl)-7, 8, 9, 10-tetrahydro-benzo[*a*]pyrene
CIA: chemiluminescence immunoassay
CT: cytotrophoblast
HPV: Human Papillomavirus
IHC: immunohistochemistry
LOD: limit of detection
OD: optical density, or mean color intensity reported by the ACIS
PAH: polycyclic aromatic hydrocarbon
PhIP: 2-amino-1-methyl-6-phenylimidazole[4,5-b]-pyridine
PZ: peripheral zone of the human prostate
ST: syncytiotrophoblast
TZ: transition zone of the human prostate
VIN: Vulvar Intraepithelial Neoplasia
